# Allocation techniques for balance at baseline in cluster randomized trials: a methodological review

**DOI:** 10.1186/1745-6215-13-120

**Published:** 2012-08-01

**Authors:** Noah M Ivers, Ilana J Halperin, Jan Barnsley, Jeremy M Grimshaw, Baiju R Shah, Karen Tu, Ross Upshur, Merrick Zwarenstein

**Affiliations:** 1 Family Practice Health Centre, Women’s College Hospital, 76 Grenville Street, Toronto, ON, M5S1B2, Canada; 2 Department of Family and Community Medicine, University of Toronto, 500 University Avenue, 5th Floor, Toronto, ON, M5G1V7, Canada; 3 Institute for Clinical Evaluative Sciences, 2075 Bayview Avenue, Toronto, ON, M4N3M5, Canada; 4 Institute of Health Policy Management and Evaluation, University of Toronto, Health Sciences Building, 155 College Street, Suite 425, Toronto, ON, M5T3M6, Canada; 5 Division of Endocrinology and Metabolism, Department of Medicine, University of Toronto, 200 Elizabeth St, EN12-218, Toronto, ON, M5G2C4, Canada; 6 Clinical Epidemiology Program, Ottawa Health Research Institute, 1053 Carling Avenue, Administration Building, Room 2-017, Ottawa, ON, K1Y 4E9, Canada; 7 Sunnybrook Health Sciences Centre, 2075 Bayview Avenue, Toronto, ON, M4N 3 M5, Canada; 8 Toronto Western Hospital Family Health Team, University Health Network, 399 Bathurst Street, West Wing, 2nd Floor, Toronto, ON, M5T2S8, Canada

**Keywords:** Cluster-randomized trials, Balanced allocation, Restricted randomization

## Abstract

Reviews have repeatedly noted important methodological issues in the conduct and reporting of cluster randomized controlled trials (C-RCTs). These reviews usually focus on whether the intracluster correlation was explicitly considered in the design and analysis of the C-RCT. However, another important aspect requiring special attention in C-RCTs is the risk for imbalance of covariates at baseline. Imbalance of important covariates at baseline decreases statistical power and precision of the results. Imbalance also reduces face validity and credibility of the trial results. The risk of imbalance is elevated in C-RCTs compared to trials randomizing individuals because of the difficulties in recruiting clusters and the nested nature of correlated patient-level data. A variety of restricted randomization methods have been proposed as way to minimize risk of imbalance. However, there is little guidance regarding how to best restrict randomization for any given C-RCT. The advantages and limitations of different allocation techniques, including stratification, matching, minimization, and covariate-constrained randomization are reviewed as they pertain to C-RCTs to provide investigators with guidance for choosing the best allocation technique for their trial.

## Review

### Introduction

Cluster-randomized controlled trials (C-RCTs) allocate intact social units (clusters, such as hospitals, schools, or communities) and collect data from members of those social units (individuals, such as patients, students, or citizens). Since data from individuals within a cluster cannot be assumed to be independent of each other, C-RCTs require unique methodological considerations compared to trials randomizing individuals (I-RCTs) [1]. Unfortunately, reviews have repeatedly noted important methodological issues in the conduct and reporting of cluster randomized trials (C-RCTs) [2-6]. In particular, clustering must be taken into account when calculating the sample size and conducting analyses in a C-RCT [7]. However, investigators embarking on a C-RCT must consider more than just adaptations to the sample size and the analytic plan when designing their trial. Another criterion that can impact upon judgments regarding the validity of a trial, covariate balance at baseline across treatment groups, also requires added attention in C-RCTs.

### The importance of balance at baseline

Balance at baseline in an experiment provides a foundation for causal inference by enhancing credibility of asymptotic claims that groups are equal. In an RCT, balance at baseline of measurable covariates similarly provides a theoretical basis to attribute measured effects to the intervention (or reassurance that a null finding is indeed null). Two additional reasons to seek balance at baseline are described below, first for RCTs in general and then for C-RCTs specifically.

The first additional reason to seek baseline balance across covariates is that it increases analytical power and statistical precision [8]. The chance imbalances created by simple randomization will tend to increase the variance of the estimated treatment effect and therefore decrease the efficiency of the trial [9]. Simulation studies show that a trial that uses simple randomization to allocate intervention groups risks chance imbalances that can result in a ‘loss’ of information [10]; the implication may be an important decrease in power and widened confidence intervals around the estimate of effect. Conversely, improving efficiency by increasing balance may have a non-trivial impact on reducing trial costs. Therefore, measures of imbalance have been used as a criterion to compare the efficiency of simple randomization with other allocation techniques [11]. Other ways to increase statistical power in trials include targeted patient selection and use of covariate-adjusted analysis [12], but these approaches are outside the scope of this paper.

The second additional reason to seek baseline balance is to increase the face validity, credibility, and potential impact of the trial. Even when *a priori* adjusted analyses are appropriately [13] used to analyze a randomized trial, readers may find such analyses less transparent and thus less trustworthy, especially when adjusted and unadjusted results are grossly different [9]. Therefore, even when adjusted analysis may increase power in an I-RCT, they may be avoided to simplify presentation of results.

### Balance at baseline for C-RCTs

Unfortunately, methodological reviews of C-RCTs consistently find that investigators neglect to account for correlated data when projecting the required sample size and subsequently run studies that are underpowered [5]. It is especially important to consider statistical efficiency in C-RCTs, since recruiting clusters is often more difficult than recruiting individuals [14]. Furthermore, variability in cluster size due to challenges with recruiting equal numbers of participants per cluster will exacerbate loss of power [15-17]. Although block-randomization in C-RCTs can improve balance in numbers of clusters, it does not address number of participants within clusters. (This problem may be partially addressed if the cluster size is known in advance by rank-ordering the clusters by size, then applying block-randomization [18] or by stratified participant-level recruitment, if recruitment of participants occurs after recruitment of clusters [19].) Since power in C-RCTs may be limited by the challenges of recruiting both clusters and participants and because power is further limited by variability in the number of participants within clusters, trying to retain power by ensuring balance in the baseline characteristics of both clusters and participants in C-RCTs becomes even more important. Therefore, investigators planning a cluster trial should consider options for actively balancing baseline covariates in addition to taking precautions to balance the number of participants.

C-RCTs also require special attention with respect to the perceived credibility of results. In general, analyses in C-RCTs are slightly more complex than in I-RCTs to account for clustering. It has been proposed that results based on analysis of C-RCTs from hierarchical models that properly account for both patient-level results and cluster-level data may be less transparent to readers [20]. Therefore, balance at baseline may play an especially important role in C-RCTs by reducing the difference between adjusted and unadjusted results.

### Random allocation and balance at baseline in C-RCTs

Small differences at baseline in a properly conducted I-RCT are thought to represent chance findings rather than bias [21]. Given a large enough sample, ‘simple’ (or complete) randomization is expected to produce balance in C-RCTs across cluster-level covariates because this is the level of allocation. Unfortunately, many C-RCTs do not have enough clusters to create a reasonable expectation for cluster-level balance. Consider the review of C-RCTs published between 2000 and 2008 [4], which found that the median number of clusters was 21, but 25% had fewer than 12 and 14% had less than four clusters per arm (the fewest recommended to ensure statistical validity [22]).

In C-RCTs, baseline balance is needed at the level of the individual as well as the level of the cluster. Since participants in each cluster are likely to share certain characteristics, important participant-level covariate imbalances across treatment arms are possible, even if cluster-level characteristics are fully balanced. In a large C-RCT aiming to improve management of osteoporosis, which used simple randomization to allocate 435 physicians (clusters) and 1,973 patients, sufficient balance was achieved across physician characteristics, but not for patient-level characteristics [23]. In particular, the groups had important differences in the proportion of patients with a history of a fracture (a prognostically important covariate). In a separate paper, the authors showed that improved balance at baseline would have been attainable through restricted randomization, leveraging information available to the investigators in administrative databases prior to allocation [24].

A review of 36 C-RCTs published in the *BMJLancet*, and *New England Journal of Medicine* between 1997 and 2002 found that three (8%) had evidence for cluster imbalance [25]. Two of the three trials with imbalance had four or less clusters per arm and neither used restricted randomization [26,27]. In both cases, schools were the unit of allocation and sex of the schoolchildren was an important and unbalanced confounder. One trial explicitly recognized that having more single-sex schools in one arm was the cause of this imbalance. Of note, the result of not balancing this important covariate in this case led to opposite findings between the unadjusted and adjusted results [26]. The third trial in the review noted to have evidence of cluster imbalance randomized from 10 pairs that were matched on two covariates. Unfortunately, the matching was ineffective; large differences between intervention and control arms existing for a key process variable, again resulting in confusing differences between adjusted and unadjusted results [28]. In contrast, of the 18 studies in the review judged to have adequate baseline balance, 13 (72%) used restricted randomization and of the 15 studies in the review judged to have unclear evidence of baseline balance, 12 (80%) used restricted randomization [25].

Although simple randomization may frequently be inadequate to achieve balance in C-RCTs, a review of 300 randomly selected C-RCTs published from 2000 to 2008 found that only 56% used restricted randomization and that this proportion was not increasing over time [4]. Further exploration of data from that review revealed that 19% overall used matching, 32% used stratification, and 4% used other restricted randomization strategies to achieve balance. Given that restricted randomization would be more likely to achieve baseline balance, the large minority of C-RCTs still using simple randomization may represent a gap in methodological best practices.

### Allocation techniques for C-RCTs: restricted randomization

The limited uptake of restricted randomization in C-RCTs suggests a need for investigators to become better versed in these options so that they may work with statisticians to consider advantages and limitations for their particular trial. Therefore, the advantages and limitations of some of the major strategies as they relate to C-RCTs are described below and summarized in Table 1. This is not a complete catalogue of allocation techniques, but rather an introduction to the array of options available to investigators. Since most investigators using restricted randomization techniques have relied on stratification and/or matching, emphasis is placed on other allocation strategies that are particularly promising for C-RCTs. Of these, minimization represents the prototypical option for when clusters are recruited and allocated sequentially, while covariate-constrained randomization represents an ideal option for when blocks of clusters (or all clusters) are recruited prior to allocation.

**Table 1:** Allocation techniques for covariate balance in C-RCTs: advantages and limitations

**Technique**	**Advantages**	**Limitations**
Simple/Complete randomization	No need for baseline data; most transparent, accepted	Higher risk for imbalance
Restricted randomization		
Matching	Improves face validity; May balance effectively for many covariates (only if a good match is found)	Loss to follow-up is doubled (pair instead of single loss); challenges with analysis; difficult to estimate/report ICC; reduced degrees of freedom limits power
Stratification	May be used in combination with other allocation techniques	Can balance for few covariates on its own
Minimization	Can balance effectively for many covariates	Less transparent, possibly less well-understood by audience; continuous covariates may need to be split into categories; potential for selection bias/predictability
Covariate-constrained randomization	Balances most effectively for many covariates; limits risk of selection bias	Requires access to baseline data; possibly less well-understood by audience; potential for over-constraint; requires additional statistical support; allocation must occur after recruitment

#### Matching

Many investigators conducting community intervention trials believe that matching is a useful mechanism for creating comparable groups at baseline [29]. Matching provides ‘face validity’ regarding balance between allocation arms [30] and is thought to be particularly useful when there are few clusters [31]. For example, one C-RCT matched six pairs of communities by ensuring that they shared geographical characteristics, as well as baseline rates of disease (Table 2) [32].

**Table 2:** Examples of restricted randomization descriptions from C-RCTs published in high impact journals

Matching	‘To help ensure comparability of the intervention and comparison communities with respect to baseline HIV and STD prevalence and risk factors for infection, the communities were matched into six pairs according to the following criteria: roadside, lakeshore, island, or rural location; geographical area (paired communities were generally in the same district and less than 50 km apart); and prior STD attendance rates at the health centre. In each matched pair, one community was randomly chosen to receive the STD intervention’ [32].
Stratification	‘To ensure balance between the 2 study arms, family physician practices underwent stratified randomization on the basis of the mean age (< 65 v. ≥ 65 years) and annual rates of emergency department visits (< 200 v. ≥ 200) of their clientele. Stratified randomization was achieved by a separate randomization procedure performed within each of the strata’ [33].
Minimization	‘We randomized practices to intervention and control groups using a minimization programme, stratifying by partnership size, training practice status, hospital admission rate for asthma, employment of practice nurse, and whether the practice nurse was trained in asthma care’ [34].
Covariate-constrained randomization	‘A balanced randomization procedure ensured that the intervention and control hospitals were balanced with respect to the rates of prophylactic use of oxytocin and episiotomy, the presence or absence of residency programs, the country and region where the hospital was located, and the annual number of births at the hospital. Of 184,756 possible ways of assigning hospitals to the intervention and control groups with acceptable balance, one sequence was randomly selected to determine the composition of the two groups’ [35].

However, pair-wise matching has a number of challenges in C-RCTs [36,37]. First, loss of follow-up from one cluster removes also its match from analysis - this is also true in I-RCTs, but a loss of a pair of clusters could be catastrophic for a trial with a small number of clusters. Furthermore, in C-RCTs, achieving a ‘good’ match that increases power is more difficult as the intra-cluster correlation (ICC) decreases (because as the amount of variability between groups decreases, it becomes harder for the matching process to remove substantial variability) [38]. In the process of developing matches, one creates the important disadvantage in C-RCTs of making it difficult to properly calculate the ICC [30], which should be reported to provide guidance to future investigators planning appropriately powered trials. Relatedly, matching may complicate the analysis of the C-RCT, especially when it is desirable to investigate the impact of individual-level factors on the likelihood of the outcome [39]. When the correlation between matched pairs is poor [40], or when there is a desire to determine the impact of baseline covariates on the intervention effect, investigators have pursued ‘breaking the matching’ in analysis; this approach may increase risk for type 1 error [37].

#### Stratification

Stratified randomization in C-RCTs has the same major limitation as in I-RCTs; the number of strata must be few to avoid unequal allocation (for example, due to incomplete blocks). This is because as the number of strata increase, the risk of incomplete filling of blocks also increase, thereby increasing the risk for imbalances in prognostic variables [41,42]. Simulations suggest that the when the total number of strata approach half the total number of units to be allocated (that is, clusters), stratification becomes ineffective [43] others have recommended limiting the number of strata to less than one-quarter the number of units to be allocated [42]. Since there usually exist prognostically important covariates at both the cluster and individual levels, many C-RCTs may require active balancing for more covariates than stratification could accommodate. For example, if there are two covariates composed of four and two levels, respectively (for example, region: north, south, east, west; and sex: male, female) the result is eight total strata, suggesting that least 16 (and ideally 32) clusters would be necessary to safely achieve balance.

Note also that balancing for individual-level covariates would require calculating the cluster-level mean (or median) for the variable of interest prior to stratifying. For example, one C-RCT testing an intervention directed at family physicians aimed to reduce visits to the repeat emergency department by patients (Table 2). It stratified the family physicians by a cluster-level covariate (older versus younger physicians) and by a cluster-level mean of a participant-level covariate (high *vs.* low rates of emergency department visits) [33]. In some instances, one could imagine stratifying clusters for political or practical reasons (for example, by geographical location). In such a scenario, there may be a need for additional balancing techniques when allocating within strata [44]; this should be planned with careful statistical support.

#### Minimization

Taves described minimization in 1974 [45] while Pocock and Simon independently reported its potential benefits in 1975 [41]. Scott and colleagues provide an excellent review of minimization discussing the benefits and limitations of minimization for I-RCTs [46]. In general, this technique randomly assigns the first participants, then accounts for the covariates of participants previously enrolled and assigns each new participant to the group that provides better balance. As shown in Table 3, if the seventh patient to be allocated to a trial has a high rate at baseline for the outcome of interest (for example, blood pressure) and a moderate rate for a covariate (for example, age), the computer algorithm will account for the characteristics of the six patients already allocated and assign the seventh patient to the arm that improves overall balance in those covariates.

**Table 3:** **Example of minimization (adapted from *Scott *et al. *2002*)**[46]

**Covariate**	**Intervention**	**Control**
Baseline rate		
High	2	2
Moderate	2	3
Low	1	1
Covariate rate		
High	2	3
Moderate	3	1
Low	1	1

Allocation of seventh patient with high baseline rate and moderate covariate rate.

Marginal totals if allocated to intervention group:

Baseline: |(2 + 1) - 2| = 1; Covariate: |(3 + 1) - 1| = 3; 1 + 3 = 4.

Marginal totals if allocated to control group:

Baseline: |2 - (2 + 1)| = 1; Covariate: |3 - (1 + 1)| = 1; 1 + 1 = 2.

Therefore, patient allocated to control because 2 < 4.

Minimization improves covariate balance compared to both simple randomization and stratification; the difference is greater in smaller trials, but this advantage of minimization has been shown to hold for I-RCTs until the sample exceeds 1,000 patients [46]. Simulations indicate that increasing the number of covariates in minimization does not substantially increase imbalance (in comparison to stratification) [47]. The number of covariates to be included in the minimization algorithm is primarily limited by statistical concerns since it is recommended that all covariates minimized should be included in statistical analysis [48]. The ability of minimization to balance more covariates has led to the suggestion by some commentators that it is the ‘platinum standard’ of allocation techniques [49].

The pharmaceutical industry [50] and other commentators [51] warn against the use of minimization mainly due to higher risk of selection bias that comes with predictability of deterministic assignment. The extent of this risk is debated [52] and must be weighed against the advantages of greater covariate balance. A random component may be added to the minimization procedure so that as imbalance grows the odds of allocation to the arm that reduces imbalance also grow, but are never equal to one [53]. This may have the advantage of reducing predictability of allocation. Many authors have suggested additional variations on the general minimization approach either to further improve balance or to reduce risk of selection bias [11,54-56]. For instance, Begg and Iglewicz [57] (and later Atkinson [58]) applied optimum design theory (minimizing the variance in the model relating the covariates to the outcome) and allowed for balancing of continuous variables obviating the need to categorize continuous covariates as high and low. It is unclear whether the theoretical advantages of these more complex techniques translate into practical benefit in typical trials [59].

Another concern may be that by forcing balance in known prognostic covariates, an investigator could (unknowingly) cause imbalance in unmeasured factors. However, it has been suggested that the balance for unmeasured variables can be no worse due to minimization because whenever the unmeasured factor is correlated with the minimized covariate, the balance for this factor will actually be improved and whenever the unmeasured factor is not correlated at all with the minimized covariate then its distribution would be unaffected [60]. Although it cannot be proven empirically without measuring the unmeasurable, this explains why balance of non-targeted variables should not made worse by using minimization [61].

The ability of minimization to balance many covariates within a small trial should make it a particularly good allocation technique for C-RCTs. However, to actively balance numerous covariates requires access to data at the time of recruitment; cluster-level means (or medians) would be used to minimize participant-level covariates, such as the practice-level mean of patient blood pressure values [62]. Fortunately, many C-RCTs take place in the context of medical systems with administrative data or access to historical records [24]. Despite its promising features and the availability of free software to implement it [63], minimization was used in only 2% of 300 randomly selected C-RCTs published from 2000 to 2008 [4]. (Although it is possible that this is an underestimate if minimization is misreported as stratification, this is similar to the estimated overall proportion of trials that use minimization [48].) One C-RCT using minimization was published in the *BMJ* in 2004 (Table 2). It allocated 44 GP practices (clusters) minimizing imbalance across four covariates with 54 total strata [34]. This trial tested a nurse outreach model aiming to support primary care providers in caring for patients with asthma. It was important to achieve balance across multiple cluster and individual level variables that might confound the effect of the intervention on asthma emergency visits. If the investigators had used traditional stratification, the trial would have been at high risk of imbalance due to over-stratification.

#### Covariate-constrained randomization

If data are available for the important cluster and/or individual-level covariates of participants prior to the allocation procedure, more complex techniques may be used to ensure acceptable balance. For example, Moulton [64] described a procedure in which a statistical program could be used to enumerate all the possible allocations of participating clusters when clusters and their covariates are known in advance. Next, the investigators would narrow this list of allocations down to the ones that met prespecified criteria for balance across baseline covariates. Finally, the actual allocation would be chosen randomly from this constrained list, thereby achieving an acceptable allocation while retaining randomness in the selection process. As seen in Table 4, if there were only four clusters recruited, these could be allocated into two arms in six different ways. In two of the possible allocations (A, F), the difference in the baseline performance is very large. In a trial with more clusters, the possible allocations increases exponentially, and it is possible to remove unacceptable allocations from the list and chose randomly from any remaining allocations with acceptable balance.

**Table 4:** **Example of covariate-constrained randomization (adapted from *Moulton 2004*)**[64]

	**Baseline performance**				
Allocation	Intervention		Control		Difference
A	25	50	60	75	30
B	25	60	50	75	20
C	25	75	50	60	5
D	50	60	25	75	5
E	50	75	25	60	20
F	60	75	25	50	30
	**Covariate rate**				
Allocation	Intervention		Control		Difference
A	80	60	75	70	2.5
B	80	75	60	70	12.5
C	80	70	60	75	7.5
D	60	75	80	70	7.5
E	60	70	80	75	12.5
F	75	70	80	60	2.5

A to F each represent different possible allocations for four clusters showing absolute difference between arms for mean rate of baseline performance and the mean rate of one additional covariate.

This approach has been shown in simulation studies to provide even better balance than minimization resulting in increased power, especially for trials with few units allocated as is common in C-RCTs [65,66]. This may be partially explained by the fact that covariate-constrained randomization can balance continuous covariates without loss of power from categorization of these variables (for example, high, medium, low) as occurs in minimization. However, when there are very few clusters as in the example illustrated in Table 4, the parameters for assessing balance may need to be widened so that the actual allocation to be utilized can be randomly selected from a larger set. Over-constraint due to strict balancing requirements that result in very few eligible allocations may force certain clusters together. For example, in Table 4, if the caliper for balance in the baseline rate or the confounder rate was set at a mean difference of 10, only two allocations would remain and both feature the highest and lowest ranking clusters together in one arm. This is not desirable since it means that the allocation is no longer truly random and this situation may invite skepticism regarding active manipulation by the investigator [64]. In addition to requiring added statistical support during the process of recruitment and allocation, the main drawback of this approach is that to acquire the necessary data, recruitment of numerous clusters must be completed prior to any cluster allocation. Investigators can allocate blocks of clusters as they are enrolled, though the first block should have at least eight units and the subsequent ones at least six [67].

Covariate-constrained randomization was used in only 2% of 300 randomly selected C-RCTs published from 2000 to 2008 [4]. However, the availability ready-made algorithms to implement this approach [67,68] may make the process more accessible. Like minimization, it is possible to apply more complex formulae when conducting this procedure. Rather than setting parameters regarding covariates to be within 10% of each other, investigators can balance with the goal of minimizing variance or decreasing the effects of adjusted analyses in a cluster-trial, as proposed by Raab and Butcher [20]. This particular approach was used in a study published recently in the *New England Journal of Medicine*[35], indicating the growing acceptability of this allocation technique by editors (and readers).

### Choosing an allocation technique

**Figure 1:**
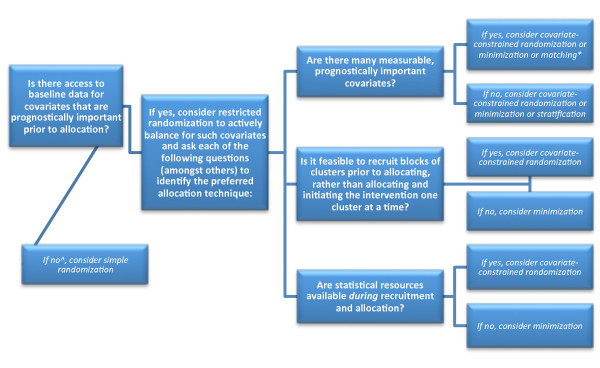
**Questions to ask and potential answers when trialists and statisticians work together to consider allocation techniques for balancing covariates in cluster-trials.**^a^One would expect that in most trials access to some relevant data would become accessible immediately after recruitment and prior to allocation. ^b^Only use matching if confident in ability to achieve a good match

Given the above considerations, investigators planning a C-RCT are encouraged to consider alternatives to simple randomization, especially when there are few total clusters (to achieve balance at the cluster-level) and/or many participants per cluster (to achieve balance at the participant level). It is also possible to combine techniques and even to actively balance participants separately from clusters (for example, by using stratified recruitment strategies if recruitment of participants can occur separately from allocation of clusters [19]). Table 1 describes the advantages and limitations various allocation techniques with respect to their potential utility for improving balance at baseline. In Figure 1, a series of questions and answers are described that may aid investigators in determining the approach most appropriate for their particular trial. In general, matching has the fewest advantages as compared to other restricted randomization options and covariate-constrained randomization seems the most favorable choice. The input of a statistician should play a key role in determining the risk of imbalance and deciding upon an allocation technique, keeping in mind that different approaches will have varying requirements for statistical support and may require unique analytic plans.

Consider, for example, our C-RCT in which two different quality improvement interventions were tested across 14 primary care clinics, aiming to improve management of patients’ blood pressure, cholesterol, and glycemic control [62]. With only 14 clusters, it seemed probable that the baseline values for the outcomes of interest (patients’ mean blood pressure, cholesterol, and glycosylated hemoglobin values) would be imbalanced. In this trial, baseline data were available, meaning that any restricted randomization technique may have been considered. Given that numerous variables were available, including baseline values for the primary outcomes and size, stratification alone was ruled out. Matching was deemed undesirable due to the risk of double-loss to follow up and challenges with analysis. Although covariate-constrained randomization was preferred given its superior ability to achieve balance, [66] it was deemed infeasible because there were outside pressures to provide the interventions immediately upon recruitment, rather than allocating blocks of clusters. As a result, minimization was selected.

In other scenarios, additional factors to consider that may have a bearing on the preferred allocation technique include: desire for a specific allocation ratio (as this would require adaptations to typical allocation techniques [56]) desire for stakeholders to witness the allocation process (as was done with one study using covariate-constrained randomization [69]); and pragmatic issues that may arise due to geographical spread of clusters and/or challenges with recruitment [15,19]. Regardless of the choice made, efforts should be made to achieve (and report) allocation concealment to limit selection bias; this is important for all trials, but especially so when deterministic rather than random allocations are used, such as minimization [51,70]. In addition, with simple or complex allocation techniques, the entire trial may be compromised if the computer program is not reliable, as happened with one large study using minimization [71].

## Conclusion

Achieving balance at baseline using simple randomization in C-RCTs is less likely than in typical RCTs due to the correlated nature of nested data and is less likely when there are few clusters to be randomized. Therefore, investigators planning C-RCTs should avoid using simple randomization, especially when there are few clusters in the trial. Given the risk of baseline imbalances for C-RCTs, the known limitations of stratification and matching, and the potential benefits of covariate-adaptive allocation techniques, investigators should consider use of the latter methods whenever important covariates are measurable prior to group assignment. In particular, when baseline data and statistical support are available and numerous clusters can be recruited prior to allocation, covariate-constrained randomization can offer investigators the chance to remove the risk of baseline imbalance with minimal risk for bias.

## Competing interests

The authors declare that they have no competing interests.

## Authors’ contributions

NI drafted the manuscript. All authors contributed to the conceptual development of the review. All authors contributed to critically revising the intellectual content of the manuscript. All authors have approved the final version.
